# A case of unexpectedly difficult intubation caused by a large asymptomatic choanal polyp

**DOI:** 10.1186/s40981-016-0028-7

**Published:** 2016-01-15

**Authors:** Yoko Hori, Kana Taniguchi, Tadashi Okabe, Atsuhiro Sakamoto

**Affiliations:** 1 Department of Anesthesiology, Nippon Medical School, Sendagi 1-1-5, Bunkyo-ku, Tokyo 113-8603 Japan; 2 Department of Anesthesiology, Hitachi, Ltd. Hitachinaka General Hospital, 20-1 Ishikawa-cho, Hitachinaka-shi, Ibaraki 312-0057 Japan

**Keywords:** Difficult intubation, Choanal polyp, Video laryngoscope

## Abstract

We report a case of unexpectedly difficult intubation in a patient with a huge but asymptomatic choanal polyp. A 77-year-old man with invasive bladder cancer was scheduled for total cystectomy under general anesthesia. However, tracheal intubation with a Macintosh laryngoscope proved impossible due to obstruction by a large oropharyngeal tumor. Using a video laryngoscope, intubation was successfully achieved. Choanal polyps are not uncommon, but large choanal polyps reaching the oropharynx appear relatively rare. Anesthesia and airway management for large oropharyngeal tumor has not been sufficiently discussed.

## Background

The prevalence of nasal polyps is reportedly 1–4 % in the United States and Europe, according to a questionnaire survey [1]. Choanal polyps are benign, unilateral masses usually originating from the mucosa of the maxillary sinus [2]. The main symptoms in patients with a choanal polyp are nasal obstruction, rhinorrhea, snoring, and nasal discharge [3]. Treatment to prevent the recurrence of choanal polyps involves complete removal of the base in the maxillary sinus mucosa and nasal polyp surgery using an endoscopic approach [4, 5].

Choanal polyps are relatively common, but large choanal polyps reaching the oropharynx are rare. Large polyps are infrequently reported in the field of otolaryngology as causes of airway obstruction [6–8], but no reports have been described in the field of anesthesiology. Airway obstruction after general anesthesia represents a dangerous situation for anesthesiologists. We report a case of unexpectedly difficult intubation in a patient with a large but asymptomatic choanal polyp, and discuss the issues of anesthesia and airway management for such cases.

## Case presentation

A 77-year-old man (height, 163 cm; weight, 57 kg) was diagnosed with invasive bladder cancer and scheduled for total cystectomy and ileal conduit diversion under general-epidural anesthesia. Preoperatively, the patient showed no findings suggesting a difficult airway; airway examination revealed mouth opening to 4 cm, full range of neck movements, and Mallampati grade II. An epidural catheter was placed at the L2-3 epidural space. After preoxygenation with 100 % oxygen, general anesthesia was rapidly induced using 0.1 mg of fentanyl and 120 mg of propofol. Manual bagmask ventilation was easily achieved after loss of consciousness, and 50 mg of rocuronium was administered.

**Fig. 1:**
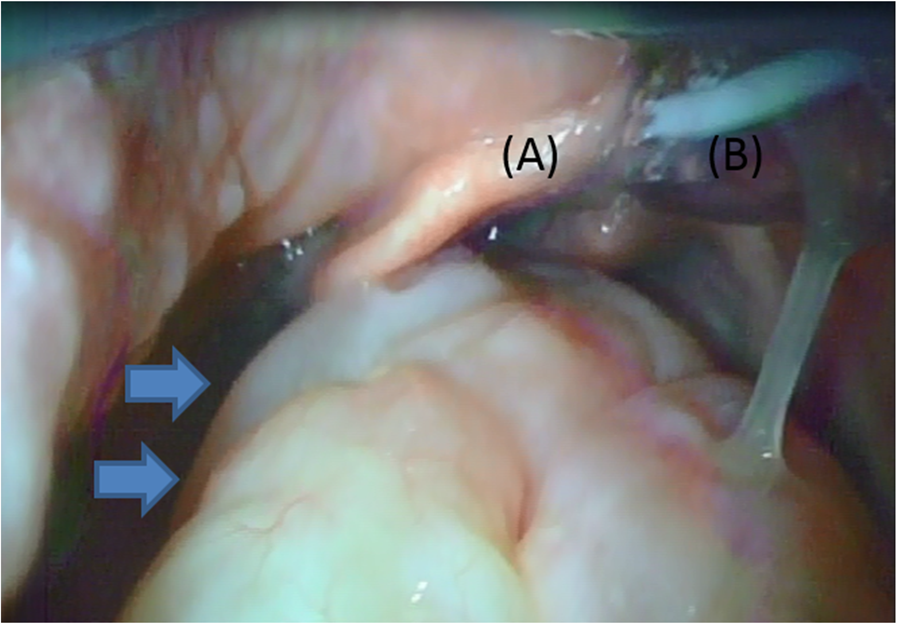
Oropharyngeal tumor. A large, roundish oropharyngeal tumor blocked the field of view during intubation. **a** Epiglottis. **b** Trachial Tube. Arrow: Tumor

We attempted to place a tracheal tube using a Macintosh laryngoscope, but unexpectedly, this proved impossible because of an obstacle, a large and roundish oropharyngeal tumor in the field of view (Fig. 1). Macintosh laryngoscopy revealed only epiglottis (Cormack-Lehane grade 3). We then used a video laryngoscope (King Vision®; King Systems, Noblesville, IN) and achieved successful intubation.

Anesthesia was maintained using 2 % sevoflurane, remifentanil at 0.05 μg/kg/min, and 120 mg of rocuronium in total. Five milliliters of 0.375 % ropivacaine was administered through the epidural catheter, and continuous dosing with 1.5 % ropivacaine was provided by epidural infusion. The operation was completed safely. We performed extubation after confirming sufficient spontaneous breathing and response of the patient, as is usually done.

**Fig. 2:**
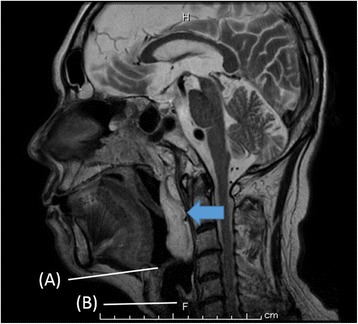
Mass filled within the right nasal cavity to the nasopharynx (MRI scans on head). Arrow: Tumor. **a** Epiglottis. **b** Trachea

After surgery, we performed magnetic resonance imaging of the head to diagnose the oropharyngeal tumor. A giant mass was seen attached to the posterior ethmoid sinus, extending from the right intranasal cavity to the nasopharynx. We diagnosed the oropharyngeal tumor as a large choanal polyp (Fig. 2).

Polypectomy was performed under general anesthesia another day. We again successfully performed tracheal intubation with the King Vision® video laryngoscope. The operation was completed without any other adverse events, and a choanal polyp measuring 14 cm in maximum diameter was removed. The definitive diagnosis from histopathological examination was choanal polyp.

In this case, the patient showed no symptoms of airway obstruction or dysphagia in preoperative interviews. Moreover, no abnormality in the oral cavity was apparent on ocular inspection. We thus could not have predicted the difficult intubation preoperatively. Fortunately, mask-ventilation proved easy, and intubation was safely achieved by displacing the mass under video laryngoscope.

Airway obstruction after extubation was a concern. After considering that the patient had no symptoms of airway obstruction preoperatively, and no injury to the mass or intraoral bleeding with the intubation maneuver, we judged the risk of airway obstruction as low and performed extubation as usual. Neither airway obstruction nor respiratory symptoms occurred after emergence from anesthesia.

Large choanal polyps have been reported in the otolaryngology area, albeit rarely, but details of anesthesia and airway management for large oropharyngeal tumors have not been sufficiently discussed [7]. In our case, mask-ventilation fortunately proved easy, probably because the structure of the choanal polyp hanging down from the choana made airway obstruction unlikely. On the other hand, another report has described a difficult airway with a different kind of oropharyngeal polyp [8], and attention to airway management is warranted in these cases.

## Conclusion

We reported a case of unexpectedly difficult intubation in a patient with a large but asymptomatic choanal polyp. Intratracheal intubation with a Mactosh laryngoscope proved impossible, and intubation was successfully achieved using a video laryngoscope.

## Consent

Written informed consent was obtained from the patient for publication of this case report and the accompanying images.
